# A genomic perspective to assessing quality of mass-reared SIT flies used in Mediterranean fruit fly (*Ceratitis capitata*) eradication in California

**DOI:** 10.1186/1471-2164-15-98

**Published:** 2014-02-05

**Authors:** Bernarda Calla, Brian Hall, Shaobin Hou, Scott M Geib

**Affiliations:** 1 Tropical Crop and Commodity Protection Research Unit, USDA-ARS Pacific Basin Agricultural Research Center, Hilo, HI 96720, USA; 2 Department of Plant and Environmental Protection Sciences, College of Tropical Agriculture and Human Resources, University of Hawai’i Manoa, Honolulu, HI 96822, USA; 3 University of Hawai’i Manoa, Honolulu, HI 96822, USA

**Keywords:** Medfly, *Ceratitis capitata*, RNA-seq, Sterile insect technique, Irradiation, Sterilization

## Abstract

**Background:**

Temperature sensitive lethal (*tsl*) mutants of the tephritid *C. capitata* are used extensively in control programs involving sterile insect technique in California. These flies are artificially reared and treated with ionizing radiation to render males sterile for further release en masse into the field to compete with wild males and disrupt establishment of invasive populations. Recent research suggests establishment of *C. capitata* in California, despite the fact that over 250 million sterile flies are released weekly as part of the state’s preventative program. In this project, genome-level quality assessment was performed, measured as expression differences between the Vienna-7 *tsl* mutants used in SIT programs and wild flies. RNA-seq was performed to provide a genome-wide map of the messenger RNA populations in *C. capitata*, and to investigate significant expression changes in Vienna-7 mass reared flies.

**Results:**

Flies from the Vienna-7 colony showed a markedly reduced abundance of transcripts related to visual and chemical responses, including light stimuli, neural development and signaling pathways when compared to wild flies. In addition, genes associated with muscle development and locomotion were shown to be reduced. This suggests that the Vienna-7 line may be less competitive in mating and host plant finding where these stimuli are utilized. Irradiated flies showed several transcripts representing stress associated with irradiation.

**Conclusions:**

There are significant changes at the transcriptome level that likely alter the competitiveness of mass reared flies and provide justification for pursuing methods for strain improvement, increasing competitiveness of mass-reared flies, or exploring alternative SIT approaches to increase the efficiency of eradication programs.

## Background

The Mediterranean fruit fly, *Ceratitis capitata* (Wiedemann) is a polyphagous insect of great economic importance. Native to Africa, this fly is now distributed worldwide, with established populations in southern Europe, the Mediterranean region, Africa, Australia and some South American countries [1]. *C. capitata* was introduced to Hawaii in 1907 and considered established by 1910 [2,3]. It represents a major and ongoing threat to the continental United States, particularly in the agriculturally rich regions of Florida and California. Each year, detections of *C. capitata* are made, and efforts are ongoing to eliminate invasive populations in order to prevent further establishment and spread in the mainland. For example, each week, over 250 million sterile male mass reared flies are released in California to disrupt establishment of invasive populations. Despite this effort, a recent analysis of historical capture patterns suggested that *C. capitata*, along with other tephritid pests, is already established in California [4]. Establishment of *C. capitata* in areas despite mass reared sterile males release suggests a weakness or failure of this approach, possibly rooted in the quality of the mass reared flies.

The sterile insect technique (SIT) is a very important and useful tool for the control of the Mediterranean fruit fly. It makes use of laboratory reared sterile males which are released in large numbers to compete with wild invasive males for fertilizing females. In rearing flies for SIT programs, the presence of females in release colonies is undesirable as it reduces the number of males that can be reared from a set amount of diet. Additionally, release of female flies along with sterile males would confound the SIT approach through additional fruit damage caused by oviposition, and through competition with wild females for mating with the sterilized male flies [5-7]. For these reasons, to improve the efficiency and reduce the cost of SIT implementation, several genetic sexing strains (GSS) were developed that allow for the selection or elimination of females early in the rearing process. GSS traits include pupal color, differential development time, and temperature sensitivity [6]. Current GSS systems in *C. capitata* are based on a translocation that links the selectable trait with the Y sex chromosome. Among these GSS, the temperature sensitive lethal mutants (*tsl*) allow for the elimination of female eggs through incubation at elevated temperature. The *tsl* trait is often linked with a white pupal trait (*wp*), which allows for straightforward visual confirmation of presence of the trait during the pupal stage. Mutants carrying these traits have been developed by researchers at the International Atomic Energy Agency in Vienna Austria (IAEA) and thus the *C. capitata tsl* strains are named “Vienna”; several independent lines have been developed from independent transformation events, with newer lines attempting to reduce trait breakdown by creating breakpoints closer to the sexing traits (e.g. Vienna-7 and Vienna-8) [5,8].

An important factor that determines the success of SIT is the competitiveness of the mass-reared sterile males with the wild males. Sterilization of males is achieved by gamma-irradiation in doses up to 145 Gy (http://www.cdfa.ca.gov/plant/pdep/prpinfo). Irradiated males are known to be at a mating disadvantage in terms of attractiveness to wild females, courtship behavior, or acceptance of the courtship by females [9,10]. The process of irradiating flies may not only render fly males sterile, but ionizing radiation may potentially cause multiple unknown mutations in the genome. Additional disadvantages in fly quality can arise from long term inbreeding of colonies reared on highly artificial environments [11]. Insects growing in captivity for several generations would likely adapt to confinement conditions and experience inbreeding depression through changes in the frequencies and allele state of mutations present in the population, reducing the genetic diversity and fitness in the wild [12-14]. Other factors such as shipping to the target program location and handling of irradiated pupae may also reduce the quality of flies.

Together, these factors bring to question the overall efficiency of mass-reared SIT flies, and demonstrate the importance of investigating differences between mass reared flies and their wild counterparts from alternative perspectives. By cataloguing and quantitatively assessing these differences at the genome and transcriptome levels, measurable change can be determined, helping to understand why mass reared flies may be outperformed in the wild. In the present work the transcriptome of *C. capitata* was constructed from adult and pupal males, and the transcript expression profiles were compared between a wild *C. capitata* strain found in Hawaii and the mass-reared GSS Vienna-7 utilized in the Mediterranean Fruit Fly Exclusion Program by the California Department of Food and Agriculture (CDFA), reared in their facility in Waimanalo, Hawaii. Analyses were done with the aim of providing a basic landscape of *C. capitata* transcriptome, as well as to shed light on the effects of long term mass rearing of the Vienna-7 line, as well as effects of gamma-irradiation. The hypothesis is that through long-term mass rearing, selection, and irradiation, there will be consistent changes in expression patterns in Vienna-7 derived flies that are indicative of reduced quality of these flies when compared to their wild counterparts. Overall, comparative analysis of expression and genetic changes that arise through mass rearing will provide insight into developing more competitive mass reared flies in SIT programs.

## Results

### Sequencing and quality filtering

For RNA-seq analysis, approximately 190 million paired 76 bp reads were obtained from Illumina Hiseq 2000 sequencing, totaling over 28 Gb of data (Table 1). These reads were evenly distributed between all of the libraries sequenced. All raw reads were submitted to the NCBI Sequence Read Archive under accession numbers SAMN02208095 – SAMN02208112 associated with BioProject PRJNA208956. From 190,186,205 raw read pairs, filtering and normalization reduced the read abundance to 17,217,414 (9.05%) reads used as input into the Trinity assembly, greatly reducing the computational requirements for assembly and avoiding complicating de Bruijn graphs with low quality or overabundant sequences.

**Table 1:** Number of raw reads obtained from samples obtained from the Illumina GAIIx sequencing

**Sample**	**Rep**	**Number of raw read pairs**	**Number of reads mapped to filtered transcriptome (%)**
Vienna7 adults, γ-irradiated	1	13,192,327	6,136,921 (46.52)
Vienna7 adults, γ-irradiated	2	9,595,143	5,075,214 (52.89)
Vienna7 adults, γ-irradiated	3	11,360,677	5,236,787 (46.10)
Vienna7 pupae, γ-irradiated	1	10,582,671	5,035,268 (47.58)
Vienna7 pupae, γ-irradiated	2	9,548,323	4,830,945 (50.59)
Vienna7 pupae, γ-irradiated	3	11,750,225	5,906,277 (50.27)
Vienna7 adults	1	11,893,729	5,540,467 (46.58)
Vienna7 adults	2	9,207,645	4,767,827 (51.78)
Vienna7 adults	3	11,887,697	6,077,674 (51.13)
Vienna7 pupae	1	13,700,496	7,150,689 (52.19)
Vienna7 pupae	2	5,836,933	3,234,876 (55.42)
Vienna7 pupae	3	4,698,850	2,473,191 (52.63)
Wild Hawaiian adults	1	7,463,699	3,524,921 (47.23)
Wild Hawaiian adults	2	10,915,724	4,983,183 (45.65)
Wild Hawaiian adults	3	11,412,891	4,996,645 (43.78)
Wild Hawaiian pupae	1	12,312,072	5,689,915 (46.21)
Wild Hawaiian pupae	2	11,240,088	5,219,322 (46.43)
Wild Hawaiian pupae	3	13,587,015	6,175,680 (45.45)
Total		190,186,205	92,055,802

### *De novo* transcriptome assembly, assembly filtering and gene prediction

The raw assembly of the *C. capitata* transcriptome was constructed using the Trinity assembly pipeline with all filtered reads from all eighteen libraries pooled into one dataset. This assembly yielded 190,958 contigs, with an N50 contig size of 2,686 bases, 64,803 contigs greater than 1000 bp, and a transcript sum of 236.1 Mb. While not all contigs produced by Trinity were likely to represent true transcripts in *C. capitata*, this contig set was used as a starting point for defining the transcriptome present in our sample. Filtering based off of read abundance and component isoform percentage removed 135,957 sequences, leaving 55,001 remaining. Further filtering through identification of likely coding sequence based on ORF prediction identified a total of 18,919 transcripts across 10,775 unigenes, with an N50 transcript size of 3,546 bp and transcript sum of 53.00 Mb. This filtered assembly is considered a high quality transcript set, and was used for downstream analysis.

### Gene annotation

Transcripts and genes identified in the final filtered assembly were annotated using public databases as described in Methods. More than 90% of the translated transcripts had homologs in the Uniprot database (e value < 1.00E-5) (Table 2). The resulting annotated transcript sequences were submitted to NCBI as Transcriptome Shotgun Assembly SUB276938, under Bioproject (PRJNA208956).

**Table 2:** Transcript and gene annotation summary

	**From total, high-quality identified and retained transcripts (18,919)**	**From total, high-quality identified genes (10,775)**
**Proteins (Uniprot)**	17,402	9,814
**Curated Proteins (UniRef)**	14,143	6,840
**Protein families (Pfam)**	13,646	7,886
**Signal Peptides (SignalP4.1)**	2,106	1,150
**Transmembrane domains (TMHMM)**	4,598	2,005
**eggNOG**	12,292	5,828
**GO**	13,648	6,552

### Pfam abundance

From the 10,776 unigenes identified in the assembly, 7,886 had a Pfam annotation based on translated sequence. These annotated genes belonged to one of 2,711 unique Pfam families. Forty six percent of these families could be placed into a Pfam Clan, for a total of 333 unique Pfam clans. The abundance of TMM normalized reads on each protein family was calculated to assess the global transcriptome composition (Additional file 1: Table S1). The protein family most abundant across unigenes was PF00089 (trypsins), followed by PF00069 (protein kinases). The protein family with the highest read counts was PF00379, annotated as chitin-binding proteins, representing structural proteins likely part of the insect cuticle; this family was over-represented in all pupae libraries of Vienna-7 and wild type. Interestingly, the second and fifth protein families with the highest read abundance corresponded to viral sequences: PF00910 (viral RNA helicases), and PF08762 (CRPV capsid protein-like family). These viral-related families showed very high counts in the Vienna-7 libraries as opposed to the wild type flies, making for up to 43% of the normalized counts in one of the irradiated libraries.

**Figure 1:**
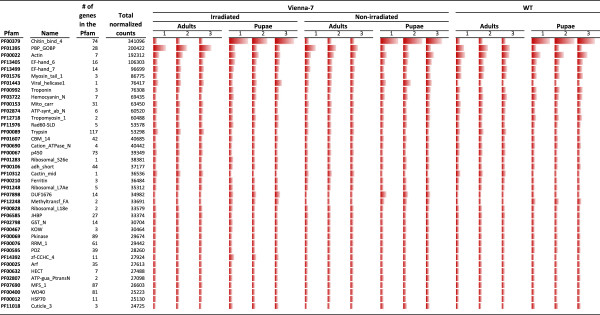
**Absolute abundance of unigenes across all libraries and total normalized counts on each library for the 40 Pfams with highest count values.** The unigenes identified in the sequencing fell into one of 2,711 unique Pfam families. TMM normalized read counts on each protein family were calculated. Viral protein families were removed to overcome expression bias. Also, a family of peptidases (PF12381) with the sixth most abundant normalized count numbers was removed as 98% of the counts were derived from a single library, clearly representing an outlier of a single replication. Chitin binding proteins were the most abundant (PF 00379) followed by a family of olfactory receptors (PF01395).

To better visualize the protein family makeup of the *C. capitata* transcriptome, the two top viral protein families were removed to overcome expression bias due to viral infection. Also, a family of peptidases (PF12381) with the sixth most abundant count numbers was removed as 98% of the counts were derived from a single library, clearly representing an outlier of a single replication. The absolute abundance of each of the top 40 Pfams is shown in Figure 1 together with the total of normalized counts for that family across all the libraries. The PBP_GOBP (PF01395), a family of olfactory receptors was the second most abundant family after chitin-binding proteins.

**Figure 2:**
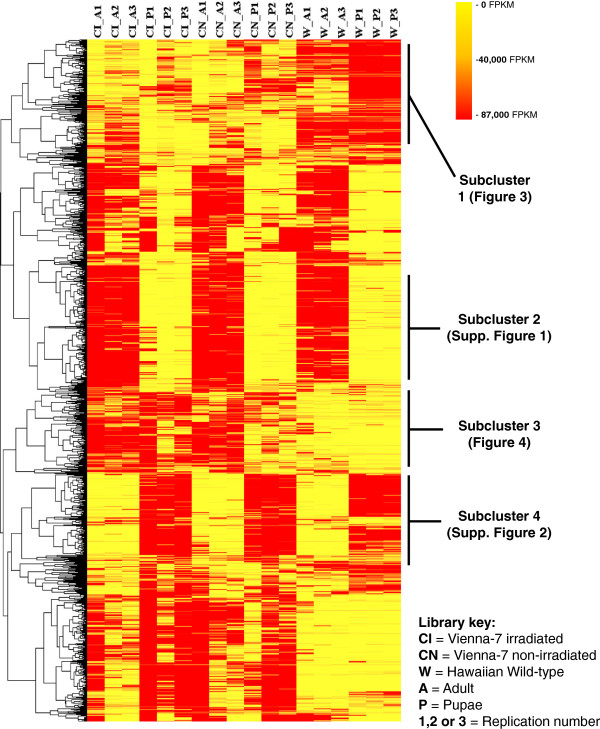
**Heatmap showing the hierarchical clustering of the 2,711 identified Pfams across the 18 sequenced libraries.** Clusters were constructed using Spearman rank-correlation coefficients on TMM-normalized counts on each of the libraries. Marked differences in normalized counts are shown between Vienna-7 and Hawaiian colonies and between developmental stages. Derived subclusters with Pfam annotations are in Additional file 2: Figures S1 and Additional file 3: Figure S2, Figures 3 and 4. Derived subclusters with Pfam annotations are presented in Additional file 2: Figures S1 and Additional file 3: Figure S2, Figures 3 and 4.

**Figure 3:**
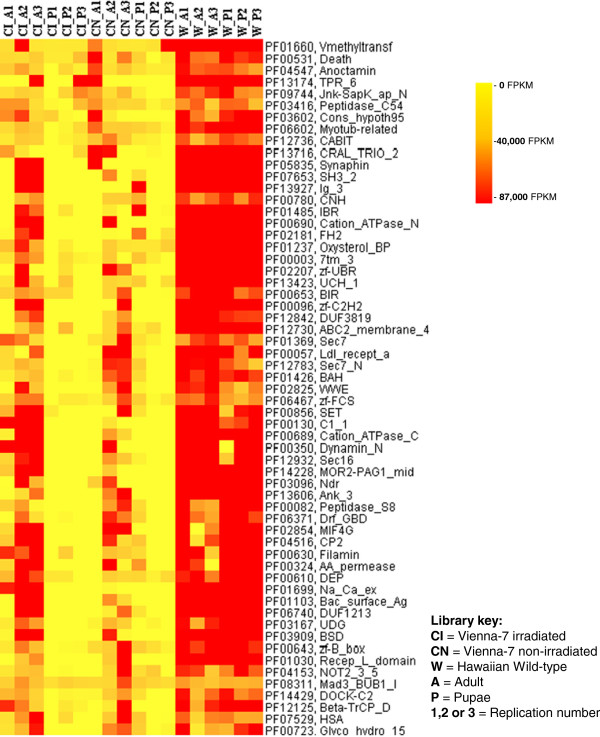
**Sub-cluster of identified Pfams derived from Figure **2**.** Differences between libraries of the Vienna-7 and the Hawaiian flies showing Pfams overrepresented in Wild Hawaiian flies.

**Figure 4:**
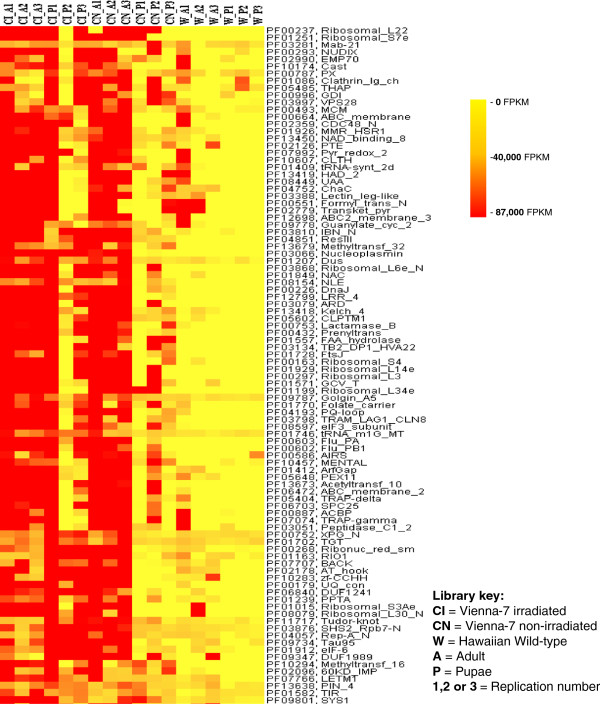
**Sub-cluster of identified Pfams derived from Figure **2**.** Differences between libraries of the Vienna-7 and the Hawaiian flies showing Pfams overrepresented in the Vienna-7 libraries.

**Figure 5:**
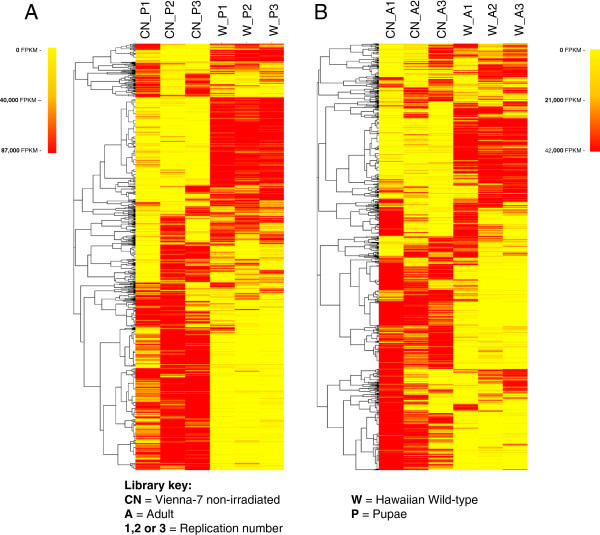
**Clustering of identified Pfams present on untreated flies from the artificially reared colony and the wild Hawaiian population.** Figure 5**a** displays pupal stage and Figure 5**b** displays adult stage. At least 50% of the protein family composition differs in abundance between both types of flies at both stages tested.

**Figure 6:**
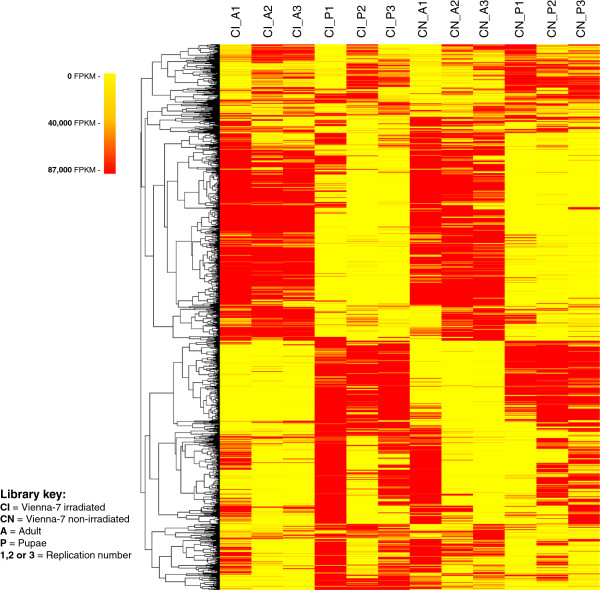
Clustering of identified Pfams in irradiated and non-irradiated fly libraries (Vienna-7 flies only); Most of the segregation was observed between the growing stages, while differences between irradiated and non-irradiated samples were minimal and mostly concentrated in the pupal stage.

A cluster based on Spearman rank-based correlation coefficients was created to group similarly expressed protein families (Figure 2). The heatmap representing the clustering showed marked grouped differences between the wild Hawaiian colony and Vienna-7. Differences between adults and pupae were also visible and perhaps more abundant, these subclusters are shown in Figure 1. Derived subclusters with Pfam annotations are presented in Additional file 2: Figure S1 and Additional file 3: Figure S2, Figures 3 and 4. A second clustering was run using only untreated flies from both the artificially reared colony and the wild Hawaiian population at either adult or pupal stage. These clusters show that at least 50% of the protein family composition differs in abundance between both types of flies at both stages tested (Figure 5a and b). Finally, irradiated and non-irradiated fly libraries (Vienna-7 flies only) were clustered together, using the same algorithm; most of the segregation was observed between the growing stages, while differences between irradiated and non-irradiated samples were minimal and mostly concentrated in the pupal stage (Figure 6). In addition, in this last clustering, we observed that the differences in protein family abundance between irradiated and non-irradiated samples were inconsistent across replication, pointing to the randomness of the irradiation effects on the genome.

## Differential gene expression

### Fold change

A statistically based measurement is necessary to identify the most potentially differentially regulated genes between repeated sequenced mRNA libraries. The overall differences between the two types of flies (Wild vs. Vienna-7), between adults and pupae, and between irradiated and non-irradiated flies from the Vienna-7 colony were tested. For these three effects a total of 5,619 unigenes were identified as statistically differentially regulated in at least one of the comparisons (FDR corrected p-value < 0.05). Not surprisingly, the comparison between adults and pupae yielded the highest number of differentially regulated transcripts (4,634 genes), followed by the comparison between the wild and Vienna-7 (968 genes). The comparison between irradiated and non-irradiated flies showed only 17 genes significantly differentially regulated. Additionally, six independent pair wise comparisons between replicated libraries were tested (Table 3); each of these yielded a range of differentially expressed genes, varying from 148 genes (Vienna-7 irradiated pupae vs. non-irradiated pupae) to over four thousand genes (wild adult vs. wild pupae).

**Table 3:** Number of declared significantly differentially regulated genes in sample pairwise comparisons

**Comparison tested**	**# of significantly differentially regulated (FDR pval <0.05)**
Vienna7 vs. Wild Hawaiian wild type (overall)	968
Adults vs pupae (overall)	4,634
Irradiated flies vs. non-irradiated flies	17
Wild Hawaiian adults vs. wild Hawaiian type pupae	4,345
Vienna7 adults vs. Vienna7 pupae	3,360
Vienna7 pupae vs. wild Hawaiian type pupae	3,094
Vienna7 adults vs. wild Hawaiian type adults	1,694
Vienna7 irradiated adults vs. Vienna7 non-irradiated adults	364
Vienna7 irradiated pupae vs. Vienna7 non-irradiated pupae	148

### Gene ontology term enrichment of differentially expressed genes

Gene Ontology (GO) classification coupled with term enrichment analysis was used to group statistically differentially regulated genes by biological function allowing for a better understanding of the biological differences between the flies used in the experiment. The term enrichment results for the comparison between adults and pupae showed highly significant enrichment on up-regulated genes with terms related to primary metabolism, including amino acid, carbohydrate, lipid and general anabolism and catabolism processes (e.g. GO:0044710, GO:0019752, GO:1901564, GO:1901605, GO:0044723, GO:0032787). Genes with down-regulation in the adults vs. pupae comparison (or with more abundance in pupae) showed very high term enrichment in cell adhesion processes (i.e. GO:0007155, GO:0022610, GO:0016337, GO:0007156, GO:0016339) and development including general anatomical, respiratory, and epithelium development (e.g. GO:0048856, GO:0007424, GO:0044767, GO:0060541, GO:0048731, GO:0030036, GO:0060429, GO:0048569). The top 40 up- and down- regulated GO terms are shown in Additional file 4: Table S2 with their respective expected values and significance p-values.

The term enrichment for the comparison between flies from the translocated Vienna-7 flies vs. wild-type flies showed an over-representation of nucleic acid processes in the artificially reared Vienna-7 (e.g. GO:0090304, GO:0006259, GO:0006139, GO:0090305, GO:0034660) Also, terms related to viral life cycle were present in this group (i.e. GO:0019079, GO:0019058 and GO:0019058). Signaling, sensory, and neurological-related processes were more abundant in the wild-type colony than in Vienna-7 (e.g. GO:0030182, GO:0050877, GO:0048699, GO:0007423, GO:0023052) (Table 4). To better understand the differences between colonies, GO term enrichment was also run on the list of significantly differentially regulated unigenes obtained from the separate pair wise comparison between Vienna-7 pupae only and wild pupae only (3,094 unigenes) and between Vienna-7 adults only and wild adults only (1,649 unigenes). Vienna-7 pupae compared to wild pupae showed several stress-related terms with higher abundance in the Vienna-7 flies, including response to cold (GO:0009409, GO:0009631), disease response (GO:0009617), and oxidative stress (increase in antioxidant machinery in response to reactive oxygen species originated by stress)-related terms (such as electron transport and oxidative phosphorylation: GO:0022900, GO:0022904, GO:0042773, GO:0006119, and GO:0042775). In addition, several viral-related terms were present, four of them in the top 40 most significant terms, with an additional “gene silencing” term (GO:0019079, GO:0019058, GO:0016032, GO:0022415, GO:0031047). Terms with less abundance in the Vienna-7 pupae than in the wild corresponded to those in signaling and neurological processes (e.g. GO:0007154, GO:0023052, GO:0044700, GO:0007165, GO:0050877) (Table 5). Comparing adults between both colonies showed more abundance of a vast amount of nucleic acid metabolism, DNA replication and cell cycle-related terms in the Vienna-7 colony (Table 6). Interestingly, the wild-type colony showed a distinctly high enrichment in terms related to response to light stimuli including phototransduction processes, rhodopsin signaling, detection of visible light and the like. A term related to perception of chemical stimulus was also found among the top highly significant GO terms, suggesting impact on both the visual and olfaction systems. This last comparison also showed higher abundance of motility-related terms in the wild fly compared to Vienna-7, these terms including muscle cell differentiation, striated muscle cell development and differentiation, locomotion and motility (Table 6).

**Table 4:** Top 20 Vienna7 vs. wild Hawaiian type differentially regulated genes GO term enrichment

**Term**	**Annotated**	**Significant**	**Expected**	**Fisher exact test**	**p-value**
***Up-regulated genes***					
GO:1901360	Organic cyclic compound metabolic processes	1289	130	81.06	6.70E-11
GO:0090304	Nucleic acid metabolic process	948	105	59.61	7.10E-11
GO:0006259	DNA metabolic process	239	42	15.03	3.50E-10
GO:0006725	Cellular aromatic compound metabolism	1231	123	77.41	7.40E-10
GO:0006396	RNA processing	166	33	10.44	1.30E-09
GO:0006139	Nucleobase-containing compound metabolism	1166	117	73.32	2.20E-09
GO:0046483	Heterocycle metabolic process	1212	120	76.21	2.80E-09
GO:0044260	Cellular macromolecule metabolic process	1441	135	90.61	5.50E-09
GO:0090305	Nucleic acid phosphodiester bond hydroly…	78	21	4.9	5.70E-09
GO:0019079	Viral genome replication	13	9	0.82	7.70E-09
GO:0042254	Ribosome biogenesis	60	18	3.77	1.10E-08
GO:0034660	NcRNA metabolic process	74	20	4.65	1.30E-08
GO:0034641	Cellular nitrogen compound metabolic pro…	1291	123	81.18	1.90E-08
GO:0022613	Ribonucleoprotein complex biogenesis	73	19	4.59	5.70E-08
GO:0006260	DNA replication	93	21	5.85	1.60E-07
GO:0044237	Cellular metabolic process	2258	181	141.99	3.90E-07
GO:0019058	Viral infectious cycle	18	9	1.13	4.00E-07
GO:0034470	NcRNA processing	53	15	3.33	4.60E-07
GO:0006807	Nitrogen compound metabolic process	1455	128	91.49	1.30E-06
GO:0006281	DNA repair	82	17	5.16	8.90E-06
***Down-regulated genes***					
GO:0048468	Cell development	610	94	42.38	1.30E-15
GO:0048646	Anatomical structure formation	662	95	45.99	9.90E-14
GO:0051239	Regulation of multicellular organismal processes	413	67	28.69	5.00E-12
GO:0030182	Neuron differentiation	331	58	23	7.30E-12
GO:0003008	System process	462	70	32.1	4.20E-11
GO:0030154	Cell differentiation	927	112	64.41	4.60E-11
GO:0009653	Anatomical structure morphogenesis	761	97	52.87	8.80E-11
GO:0048869	Cellular developmental process	964	114	66.98	1.20E-10
GO:0050877	Neurological system process	385	61	26.75	1.60E-10
GO:0048699	Generation of neurons	356	58	24.73	1.60E-10
GO:0007267	Cell-cell signaling	264	48	18.34	1.70E-10
GO:0044767	Single-organism developmental process	1185	131	82.33	2.40E-10
GO:0048513	Organ development	697	90	48.43	2.90E-10
GO:0007423	Sensory organ development	191	39	13.27	3.20E-10
GO:0023052	Signaling	980	114	68.09	3.50E-10
GO:0044700	Single organism signaling	980	114	68.09	3.50E-10
GO:0048749	Compound eye development	122	30	8.48	3.90E-10
GO:0044707	Single-multicellular organism process	1699	169	118.04	4.10E-10
GO:0007154	Cell communication	996	115	69.2	4.50E-10
GO:0042692	Muscle cell differentiation	123	30	8.55	4.80E-10

**Table 5:** Vienna7 pupae vs. wild Hawaiian wild-type pupae differentially regulated genes GO term enrichment

**Term**	**Annotated**	**Significant**	**Expected**	**Fisher exact test**	**p-value**
***Up-regulated genes***					
GO:0006412	Translation	103	45	20.64	2.90E-08
GO:0022900	Electron transport chain	48	24	9.62	3.00E-06
GO:0019079	Viral genome replication	13	10	2.61	1.60E-05
GO:0009409	Response to cold	8	7	1.6	8.40E-05
GO:0019058	Viral infectious cycle	18	11	3.61	0.00016
GO:0006091	Generation of precursor metabolites	117	40	23.45	0.0002
GO:0009631	Cold acclimation	5	5	1	0.00032
GO:0022904	Respiratory electron transport chain	12	8	2.41	0.00058
GO:0006626	Protein targeting to mitochondrion	10	7	2	0.00086
GO:0070585	Protein localization to mitochondrion	10	7	2	0.00086
GO:0072655	Establishment of protein localization	10	7	2	0.00086
GO:0065004	Protein-DNA complex assembly	27	13	5.41	0.00093
GO:0042773	ATP synthesis coupled electron transport	8	6	1.6	0.00123
GO:0042775	Mitochondrial ATP coupled electron transport	8	6	1.6	0.00123
GO:0044237	Vellular metabolic process	2258	492	452.56	0.00136
GO:0016032	Viral reproduction	39	16	7.82	0.00211
GO:0044764	Multi-organism cellular process	39	16	7.82	0.00211
GO:0008152	Metabolic process	2745	586	550.16	0.00217
GO:0016125	Sterol metabolic process	36	15	7.22	0.0024
GO:0007017	Microtubule-based process	186	53	37.28	0.00298
GO:0006119	Oxidative phosphorylation	9	6	1.8	0.00307
GO:0006767	Water-soluble vitamin metabolic process	9	6	1.8	0.00307
GO:0034660	NcRNA metabolic process	74	25	14.83	0.00365
GO:0006839	Mitochondrial transport	21	10	4.21	0.00405
GO:0006457	Protein folding	52	19	10.42	0.00407
GO:0042254	Ribosome biogenesis	60	21	12.03	0.00467
GO:0042982	Amyloid precursor protein metabolism	7	5	1.4	0.00468
GO:0045940	Positive regulation of steroid metabolism	7	5	1.4	0.00468
GO:0031047	Gene silencing by RNA	28	12	5.61	0.00491
GO:0015931	Nucleobase-containing compound transport	53	19	10.62	0.00518
GO:0009617	Response to bacterium	72	24	14.43	0.00528
GO:0044267	Cellular protein metabolic process	609	146	122.06	0.00579
GO:0000226	Microtubule cytoskeleton organization	137	40	27.46	0.00594
GO:0022415	Viral reproductive process	32	13	6.41	0.00598
GO:0007379	Segment specification	22	10	4.41	0.00611
GO:0008202	Steroid metabolic process	54	19	10.82	0.00653
GO:0001666	Response to hypoxia	43	16	8.62	0.00663
GO:0006379	mRNA cleavage	5	4	1	0.00674
GO:0034433	Steroid esterification	5	4	1	0.00674
GO:0034434	Sterol esterification	5	4	1	0.00674
***Down-regulated genes***					
GO:0065007	Biological regulation	1938	577	429.5	5.50E-28
GO:0050794	Regulation of cellular process	1625	503	360.13	4.80E-27
GO:0007154	Cell communication	996	346	220.73	4.00E-26
GO:0023052	Signaling	980	341	217.19	8.30E-26
GO:0044700	Single organism signaling	980	341	217.19	8.30E-26
GO:0050789	Regulation of biological process	1780	533	394.48	4.40E-25
GO:0003008	System process	462	185	102.39	2.30E-20
GO:0048646	Anatomical structure formation	662	240	146.71	9.10E-20
GO:0044699	Single-organism process	2913	755	645.58	1.90E-19
GO:0044763	Single-organism cellular process	2412	653	534.55	2.50E-19
GO:0009653	Anatomical structure morphogenesis	761	265	168.65	3.80E-19
GO:0050877	Neurological system process	385	158	85.32	1.50E-18
GO:0048468	Cell development	610	222	135.19	2.20E-18
GO:0007165	Signal transduction	804	274	178.18	2.40E-18
GO:0044707	Single-multicellular organism process	1699	491	376.53	7.30E-18
GO:0044767	Single-organism developmental process	1185	369	262.62	7.60E-18
GO:0030182	Neuron differentiation	331	140	73.36	8.60E-18
GO:0032501	Multicellular organismal process	1737	497	384.95	4.10E-17
GO:0048513	Organ development	697	242	154.47	4.10E-17
GO:0010646	Regulation of cell communication	412	161	91.31	1.90E-16
GO:0035556	Intracellular signal transduction	359	145	79.56	2.90E-16
GO:0048731	System development	1025	324	227.16	2.90E-16
GO:0023051	Regulation of signaling	417	162	92.42	2.90E-16
GO:0048699	Generation of neurons	356	144	78.9	3.20E-16
GO:0007267	Cell-cell signaling	264	115	58.51	8.30E-16
GO:0007268	Synaptic transmission	181	88	40.11	9.20E-16
GO:0048856	Anatomical structure development	1301	389	288.33	1.60E-15
GO:0009887	Organ morphogenesis	316	130	70.03	2.30E-15
GO:0032989	Cellular component morphogenesis	362	143	80.23	4.50E-15
GO:0016043	Cellular component organization	1160	353	257.08	4.90E-15
GO:0050793	Regulation of developmental process	339	135	75.13	1.40E-14
GO:0007275	Multicellular organismal development	1356	398	300.52	1.90E-14
GO:0030154	Cell differentiation	927	292	205.44	3.70E-14
GO:0035637	Multicellular organismal signaling	206	93	45.65	4.60E-14
GO:0019226	Transmission of nerve impulse	203	92	44.99	4.70E-14
GO:0000904	Cell morphogenesis involved in different…	249	106	55.18	8.40E-14
GO:0009966	Regulation of signal transduction	363	140	80.45	9.10E-14
GO:0007399	Nervous system development	617	210	136.74	1.20E-13
GO:0048666	Neuron development	294	119	65.16	1.70E-13
GO:0000902	Cell morphogenesis	315	125	69.81	2.10E-13
GO:0048869	Cellular developmental process	964	298	213.64	3.00E-13

**Table 6:** Vienna7 adults vs. wild Hawaiian wild-type adults differentially regulated genes GO term enrichment

**Term**	**Annotated**	**Significant**	**Expected**	**Fisher exact test**	**p-value**
***Up-regulated genes***					
GO:0044260	Cellular macromolecule metabolic process	1441	270	190.73	7.90E-14
GO:0006396	RNA processing	166	56	21.97	3.50E-12
GO:0044237	Cellular metabolic process	2258	372	298.87	1.40E-11
GO:0022613	Ribonucleoprotein complex biogenesis	73	31	9.66	4.60E-10
GO:0043170	Macromolecule metabolic process	1839	311	243.41	5.20E-10
GO:0008152	Metabolic process	2745	425	363.33	1.40E-09
GO:0034660	ncRNA metabolic process	74	30	9.79	3.40E-09
GO:0071704	Organic substance metabolic process	2587	403	342.41	6.90E-09
GO:0042254	Ribosome biogenesis	60	26	7.94	7.30E-09
GO:0006364	rRNA processing	35	19	4.63	8.00E-09
GO:0034470	ncRNA processing	53	24	7.02	9.60E-09
GO:0016072	rRNA metabolic process	36	19	4.76	1.50E-08
GO:0044238	Primary metabolic process	2432	382	321.9	1.70E-08
GO:0090304	Nucleic acid metabolic process	948	175	125.48	1.20E-07
GO:0006457	Protein folding	52	22	6.88	1.80E-07
GO:0034641	Cellular nitrogen compound metabolism	1291	221	170.88	8.10E-07
GO:0046483	Heterocycle metabolic process	1212	209	160.42	1.20E-06
GO:0006725	Cellular aromatic compound metabolism	1231	211	162.93	1.60E-06
GO:0006139	Nucleobase-containing compound metabolism	1166	201	154.33	2.30E-06
GO:0044267	Cellular protein metabolic process	609	118	80.61	2.50E-06
GO:1901360	Organic cyclic compound metabolism	1289	218	170.61	2.90E-06
GO:0006974	Response to DNA damage stimulus	136	38	18	3.00E-06
GO:0000075	Cell cycle checkpoint	44	18	5.82	4.30E-06
GO:0031570	DNA integrity checkpoint	33	15	4.37	5.80E-06
GO:0030163	Protein catabolic process	110	32	14.56	7.30E-06
GO:0044265	Cellular macromolecule catabolic process	143	38	18.93	1.10E-05
GO:0071156	Regulation of cell cycle arrest	47	18	6.22	1.30E-05
GO:0000077	DNA damage checkpoint	28	13	3.71	1.90E-05
GO:0009451	RNA modification	18	10	2.38	2.50E-05
GO:0007050	Cell cycle arrest	58	20	7.68	2.70E-05
GO:0016071	mRNA metabolic process	122	33	16.15	2.80E-05
GO:0051603	Proteolysis involved in cellular protein…	88	26	11.65	4.00E-05
GO:0006807	Nitrogen compound metabolic process	1455	234	192.58	5.70E-05
GO:0006270	DNA replication initiation	10	7	1.32	5.70E-05
GO:0044257	Cellular protein catabolic process	90	26	11.91	6.10E-05
GO:0019079	Viral genome replication	13	8	1.72	6.30E-05
GO:0007093	Mitotic cell cycle checkpoint	31	13	4.1	7.00E-05
GO:0022403	Cell cycle phase	246	54	32.56	7.10E-05
GO:0009057	Macromolecule catabolic process	201	46	26.6	8.70E-05
GO:0010389	Regulation of G2/M transition of mitotic…	28	12	3.71	0.0001
GO:0051329	Interphase of mitotic cell cycle	73	22	9.66	0.00011
***Down-regulated genes***					
GO:0071482	Cellular response to light stimulus	14	11	1.59	9.80E-09
GO:0007603	Phototransduction, visible light	10	9	1.14	2.70E-08
GO:0016056	Rhodopsin mediated signaling pathway	10	9	1.14	2.70E-08
GO:0048646	Anatomical structure formation involved…	662	118	75.31	4.80E-08
GO:0007600	Sensory perception	142	39	16.15	6.40E-08
GO:0009583	Detection of light stimulus	29	15	3.3	9.60E-08
GO:0009584	Detection of visible light	19	12	2.16	9.80E-08
GO:0042692	Muscle cell differentiation	123	35	13.99	1.20E-07
GO:0001539	Ciliary or flagellar motility	11	9	1.25	1.30E-07
GO:0051239	Regulation of multicellular organismal processes	413	81	46.98	1.70E-07
GO:0003008	System process	462	88	52.55	1.90E-07
GO:0009581	Detection of external stimulus	38	17	4.32	2.00E-07
GO:0016059	Deactivation of rhodopsin mediated signa…	9	8	1.02	2.20E-07
GO:0022400	Regulation of rhodopsin mediated signali…	9	8	1.02	2.20E-07
GO:0009582	Detection of abiotic stimulus	39	17	4.44	3.20E-07
GO:0055002	Striated muscle cell development	86	27	9.78	4.00E-07
GO:0007602	Phototransduction	21	12	2.39	4.60E-07
GO:0055001	Muscle cell development	92	28	10.47	5.00E-07
GO:0030239	Myofibril assembly	36	16	4.1	5.10E-07
GO:0061061	Muscle structure development	174	42	19.79	9.80E-07
GO:0050953	Sensory perception of light stimulus	70	23	7.96	1.20E-06
GO:0051606	Setection of stimulus	48	18	5.46	2.10E-06
GO:0071478	Cellular response to radiation	20	11	2.28	2.40E-06
GO:0007018	Microtubule-based movement	53	19	6.03	2.40E-06
GO:0007601	Visual perception	68	22	7.74	2.80E-06
GO:0050877	Neurological system process	385	73	43.8	3.00E-06
GO:0048468	Cell development	610	104	69.39	3.70E-06
GO:0009653	Anatomical structure morphogenesis	761	124	86.57	3.80E-06
GO:0044707	Single-multicellular organism process	1699	239	193.27	4.80E-06
GO:0051146	Striated muscle cell differentiation	108	29	12.29	5.40E-06
GO:0030182	Neuron differentiation	331	64	37.65	6.90E-06
GO:0032501	Multicellular organismal process	1737	242	197.59	9.10E-06
GO:0031032	Actomyosin structure organization	44	16	5.01	1.20E-05
GO:0040011	Locomotion	380	70	43.23	1.40E-05
GO:0009416	Response to light stimulus	55	18	6.26	1.90E-05
GO:0032101	Regulation of response to external stimuli	55	18	6.26	1.90E-05
GO:0071214	Cellular response to abiotic stimulus	28	12	3.19	2.20E-05
GO:0007606	Sensory perception of chemical stimulus	56	18	6.37	2.50E-05
GO:0032989	Cellular component morphogenesis	362	66	41.18	3.70E-05
GO:0048699	generation of neurons	356	65	40.5	4.10E-05
GO:2000026	Regulation of multicellular organismal development	228	46	25.94	5.10E-05

The term enrichment for the overall comparison between irradiated and non-irradiated samples in the Vienna-7 colony showed high significance for terms related to hormonal synthesis and related compounds in up-regulated genes (i.e. GO:0016126, GO:0008299, GO:0006694), suggesting an increased activity of these pathways as a result of irradiation. On the other hand, genes with higher expression values in the non-irradiated samples resulted in a term enrichment of high significance for several viral-related terms (e.g. GO:0019058, GO:0022415, GO:0016032, GO:0)006278 (Additional file 5: Table S3). Because of our special interest in the effects of irradiation on the Vienna-7 colony, the differential expression between irradiated and non-irradiated samples from adults and pupa were separated and the GO term enrichment for these pairwise sample comparisons were analyzed. Terms related to virus/transposon sequences were found enriched only in the down-regulated irradiated vs. non irradiated pupae dataset, suggesting that irradiation somehow affects the presence of virus or transposon mobility in the Vienna-7 colony, but only when applied to pupae. Additionally, two GO terms related to reproduction were among the top 20 found down-regulated in pupae irradiated vs. non-irradiated flies (i.e. GO:0022414 and GO:0000003), perhaps pointing to the desired sterilization results expected from the treatment (Additional file 6: Table S4). Up-regulated terms in irradiated vs. non irradiated pupae samples were highly enriched for DNA repair-related mechanism (e.g. GO:0006974, GO:0006281, GO:0006302), suggesting DNA damage (Additional file 6: Table S4). In adults, irradiation effects seemed to be very random, with biological processes affected ranging from ion transport to amino acids metabolism (Additional file 7: Table S5).

## Discussion

Whole transcriptome analyses are of growing importance for the understanding of biological processes that take place in organisms of interest. We have generated a *de novo* transcriptome assembly of the pupal and adult stages of male Mediterranean fruit fly, *C. capitata,* using paired-end RNA-seq analysis. The assembly identified 18,919 transcripts and 10,775 unigenes which were annotated and used for a descriptive and comparative analysis of the mRNA composition of Hawaiian wild flies and mass reared GSS Vienna-7 flies at adult and pupae stages, before and after ionizing radiation treatment. With the data, we were able to delineate a general protein family-based composition of *C. capitata*, as well as identify specific genes and protein families present in specific libraries. In addition, we have built and made public extensive data obtained from the sequencing. This data can be used for generation of new hypotheses regarding different aspects on the biology of this important pest as well as a resource for developing assays for assessing the quality of mass reared flies.

Differences between adults and pupae across both types of flies showed that several developmental processes were significantly overrepresented in pupae as compared to the adult. Also, the four most significantly enriched GO terms were related to cell adhesion, a process which is known to play a key role in development and tissue morphogenesis [15,16]. While these results are not surprising, they provide a means of verification on the quality of the experiment and the data analysis performed.

Grouping absolute expression values by Pfam and clustering the data, we noted considerable differences between the artificially reared colonies and the wild Hawaiian flies. Furthermore, by contrasting the Vienna-7 with the Hawaiian wild flies’ relative transcript levels and statistical significance, we have identified the presence of several viral-related sequences among the sequenced transcripts, which suggests the existence of a virus in the mass reared colony; this is supported by the occurrence of several stress-related transcripts. Preliminary analyses showed that these sequences may belong to a picornavirus; however, it is possible that these transcripts are an indication of increased or activated transposon activity in the Vienna-7 colony. That the possible presence of virus is detrimental for the quality of the reared colonies needs to be tested, as several endogenous viruses have been found in different strains of *C. capitata* and are commonly found in other fruit flies [17-19]. Nonetheless, this is an interesting point to investigate and simple measures could be employed to monitor viral levels in mass reared colonies and measures put forth to reduce the impact of virus on fly quality. Additionally, by comparing the Vienna-7 colony to wild pupae and adults, the differential expression analyses showed marked down-regulation of signaling and neurological processes. In adults, two important sensory mechanisms were observed to be down-regulated in the Vienna-7 flies: light response processes and chemoreception. A third marked difference was observed in genes related to muscle development, muscle differentiation and locomotion, which were also reduced in abundance in the Vienna-7 colonies compared to the wild Hawaiian flies. Given the number and consistency of GO terms in the above mentioned categories, we hypothesize that Vienna-7 flies may have reduced fitness and competitiveness due to impaired response to light stimuli as a consequence of mass rearing in artificial conditions under low/artificial light, this impairment being reflected at the signaling (perception and signal transduction) and neuronal levels (responsive mechanism). Vienna-7 may also have reduced host and mate finding ability due to decreased chemical sensory development. In addition muscular development is diminished, reducing movement or flight ability in the flies as well as having potential impacts on longevity. As for irradiation effects, Pfam abundance clustering demonstrated the marked randomness of the irradiation effects, while few genes may be consistently affected by the treatment. This is not to say that irradiation does not have a deleterious effect on the fly, just that expression level changes are not consistent between replicate treatments. The study showed high induction of statistically differentially regulated genes in GO terms related to DNA repair mechanisms, demonstrating the DNA damage that occurs in the irradiated samples. Single and double stranded DNA damage caused by ionizing gamma radiation has been extensively reported in mammals [20-22], and is very likely one of the effects in flies. The data also showed that irradiation may somehow affect the presence of virus in pupae. One possible explanation of this is that the proportion of the population that is virus infected is weaker than other flies, and thus do not survive the impact of irradiation. In general, we can conclude that long-term artificial rearing of flies, added to the effect of ionizing radiation, may affect several specific pathways and biological processes in the fly, all of which may translate into reduced quality of individuals released for SIT.

## Conclusions

The California Department of Food and Agriculture (CDFA) reports a weekly release of approximately 125,000 sterile flies per square mile over more than 1,200 square miles by the USDA-CDFA Mediterranean Fruit Fly Exclusion Program, at an annual cost of approximately 15 million dollars for preventive purposes (http://www.cdfa.ca.gov/plant/pdep/prpinfo/pg1.html). Under that perspective, it is highly desirable that the released sterile flies are of the best possible quality and with a high rate of competitiveness in the field. The basic principles under which current SIT methods are applied at this time (long term captivity and gamma irradiation) may limit its potential utility, and alternatives to SIT or modifications to it should be considered. One such potential approach involves transgenesis, a concept already tested in *C. capitata* through the insertion of a tetracycline-repressible transactivator [23-25], and in Olive fly (*Bactrocera olea*) with the use of a dominant, female-specific lethal genetic system [26]. Another alternative is population replacement. This method has been widely studied in the control mosquito-borne diseases with insects carrying anti-pathogen genes and thus unable to transmit disease [27]. Similar approaches could be utilized to produce only male progeny from mated females, and subsequent mating with these males will continue spreading this trait, effectively allowing the wild population to serve as the rearing mechanism. Additionally, in population replacement approaches, repeated mass release of flies would not be required, and the trait is pushed through the population. Those males carrying the trait are wild derived, and thus there is not expected to be a reduced fitness cost compared to flies derived from the wild population. As the International Atomic Energy Agency has expressed its interest in developing alternatives to gamma irradiation used for SIT programs [28], recent advances in gene silencing through RNAi methods should also be considered which could induce sterilization. These alternative approaches would have reduced impact on the quality of the fly as irradiation is not performed, which has been shown to impact courtship behavior. Overall, the analysis presented here provides the foundation for beginning to evaluate mass reared flies at a genomic level, and to develop tools for better monitoring the quality of these flies over generations of colony production.

## Methods

### Sampling of *Ceratitis capitata* Vienna-7 colony and Hawaiian wild fly

*C. capitata* Vienna-7 derived mass reared flies were obtained from the California Department of Agriculture medfly mass rearing facility in Waimanalo, Hawaii. Newly formed pupae were collected (~40 ml) in triplicate before irradiation. In addition, triplicate samples were subjected to gamma-irradiation at the USDA-APHIS irradiation facility (140 Gy) following the standard methods used for mass reared flies being shipped to California for SIT release. Both irradiated and non-irradiated flies were transferred to the USDA-ARS Pacific Basin Agricultural Research Center (PBARC) for use in this study. Pools of five pupae were flash frozen in liquid nitrogen from each replicate approximately 1 day prior to adult emergence for RNA extraction and sequencing. A subset of pupae was placed in emergence cages and adult male flies were allowed to emerge. Adults were held in emergence cages under standard rearing conditions for 2 days post-emergence and then snap frozen in pools of five for each replicate. In addition, *C. capitata* infested coffee cherries were collected at Kauai Coffee (Kalaheo, HI, USA) and transferred to USDA-ARS-PBARC. Cherries were placed on a ¼ inch mesh screen elevated above sand in a fiberglass container, allowing *C. capitata* pre-pupae to emerge from the fruit and pupate in the sand. Pupae were allowed to develop until approximately 1 day prior to adult emergence. Sex of the pupae was determined by observing presence or absence of the spatulate bristle visible through the pupal cuticle. At this time, five male pupae for each replicate were snap-frozen in liquid nitrogen for RNA extraction. The remaining male pupae were put into adult emergence cages and adults were collected in the same manner as the Vienna line described above.

### RNA extraction and sequencing

Total RNA was extracted from the triplicate samples from both pupal and adult stages of each treatment (wild, Vienna non-irradiated, and Vienna irradiated) using the Qiagen RNeasy Plus Mini Kit (Qiagen Inc., Valencia, CA, USA) following the manufactures procedures with the following modifications. Approximately 30 – 50 mg of liquid nitrogen snap-frozen tissue was placed in 600 μl Buffer RLT with 1% β-mercaptoethanol and ground carefully with a disposable micropestle in a microfuge tube. This solution was then passed through a QIAshredder column and then through a gDNA Eliminator column. In addition, before final elution, on-column DNase treatments were performed to ensure full removal of genomic DNA from sample. RNA concentration and quality was assessed using a Qubit fluorometer (Invitrogen Corp., Carlsbad, CA, USA) as well as an Agilent 2100 Bioanalyzer (Santa Clara, CA, USA) following standard protocols and assays.

Each of these total RNA samples was prepared for sequencing using the TruSeq RNA Sample Preparation Kit (Illumina Inc., San Diego, CA, USA), barcoded, and all 18 libraries pooled and sequenced on a single lane of Illumina HiSeq2000 instrument at the Yale Center for Genome Analysis (West Haven, CT, USA).

### *In silico* library normalization and *de novo* transcriptome assembly

De novo reconstruction of the transcriptome was done utilizing the Trinity package [29]. Raw reads obtained were first normalized to reduce redundant read data and discard read errors using Trinity’s normalize_by_kmer_coverage.pl script with a kmer size of 25 and maximum read coverage of 30. The Trinity *de novo* RNAseq assembly pipeline (Inchworm, Chrysalis, and Butterfly) was executed using default parameters, implementing the --REDUCE flag in Butterfly and utilizing the Jellyfish k-mer counting approach [30]. Assembly was completed in 3 hours and 13 minutes on a compute node with 32 Xeon 3.1 GHz cpus and 256 GB of RAM on the USDA-ARS Pacific Basin Agricultural Research Center Moana compute cluster (http://moana.dnsalias.org).

### Assembly filtering and gene prediction

The output of the Trinity pipeline is a FASTA formatted file containing sequences defined as a set of transcripts, including alternatively spliced isoforms determined during graph reconstruction in the Butterfly step. These transcripts are grouped into gene components which represent multiple isoforms across a single unigene model. While many full length transcripts were expected to be present, it is likely that the assembly also consisted of erroneous contigs, partial transcript fragments, and non-coding RNA molecules. This collection of sequences was thus filtered to identify contigs containing full or near full length transcripts or likely coding regions and isoforms that are represented at a minimum level based off of read abundance. Pooled non-normalized reads were aligned to the unfiltered Trinity.fasta transcript file using bowtie 0.12.7 [31], through the alignReads.pl script distributed with Trinity. Abundance of each transcript was calculated using RSEM 1.2.0 (RNA-Seq by Expectation Maximization) [32], utilizing the Trinity wrapper ‘run_RSEM.pl’. Through this wrapper, RSEM read abundance values were calculated on a per-isoform and per-unigene basis. In addition, percent composition of each transcript component of each unigene was calculated. From these results, the original assembly file produced by Trinity was filtered to remove transcripts that represent less than 5% of the RSEM based expression level of its parent unigene or transcripts with transcripts per million (TPM) value below 0.5.

Coding sequence was predicted from the filtered transcripts using the ‘transcripts_to_best_scoring_ORFs.pl’ script distributed with the Trinity software from both strands of the transcripts. This approach uses the software Transdecoder (http://transdecoder.sourceforge.net/) which first identifies the longest open reading frame (ORF) for each transcript and then uses the 500 longest ORFs to build a Markov model against a randomization of these ORFs to distinguish between coding and non-coding regions. This model is then used to score the likelihood of the longest ORFs in all of the transcripts, reporting only those putative ORFs which outscore the other reading frames. Thus, the low abundance filtered transcript assembly was split into contigs that contain complete open reading frames, contigs containing transcript fragments with predicted partial open reading frames, and contigs containing no ORF prediction. The resulting retained transcript sets containing transcripts above the abundance threshold and containing a likely open reading frame were merged and subjected to annotation and utilized in subsequent analysis.

### Gene annotation

The filtered transcripts were annotated using the UniRef SwissProt database, Pfam-A, eggNOG, and gene ontology utilizing a beta release of the Trinotate annotation pipeline. The filtered transcript set was first subjected to blastp alignment against the UniRef-Swissprot database (downloaded 12/11/2012) using blast-2-2-26+ with e-value cutoff of 1.0E-5. In addition, protein domains were identified through searching the Pfam_A database using HMMER 3.0. Signal peptides and transmembrane regions were annotated with SignalP 4.1 and TMHMM 2.0, respectively. The resulting outputs were loaded into a Trinotate database where eggNOG and Gene Ontology terms were added and the resulting annotation set was exported as a delimited file for further analysis. In addition, transcripts were subjected to blastx alignment against the *Drosophila melanogaster* protein set (http://Flybase.org, Dmel-r5.44) [33,34] and UniRef90 using an e-value cutoff of 1e-5 to identify homologous genes in these databases.

### Read library mapping and expression analysis

Because the Trinity assembler is able to accurately predict splice isoforms, gene and isoform expression quantification was performed using RSEM, which is particularly well suited to work with multiple isoforms where the same read may map to multiple sequences. The filtered transcript set described above (containing transcripts passing a minimum read abundance cutoff and containing predicted full or partial ORFs) was used for analysis to avoid skewing expression quantification results with non-coding and fragmented data. Reads from each sequencing library were independently mapped to this high confidence transcriptome assembly using bowtie (v 0.12.7) using the alignReads.pl script distributed with Trinity. The resulting bam formatted mapping files were sorted and used to produce fragment abundance estimation by RSEM. Transcript abundance values were produced as expected read count at both unigene and individual transcript isoform level.

### Absolute expression analysis by Pfam

Read count values for unigenes were normalized using the trimmed mean of M values (TMM) method and transformed into fragments per feature kilobase per million reads mapped (FPKM) for each gene and the individual isoforms that compose each gene for each developmental library using scripts provided by Trinity. TMM-FPKM normalized read counts across genes in the same Pfam family were added together to assess family abundance. For clustering, Pfams with less than two gene members and normalized counts less than 50 in at least one library were removed. Pfams were clustered using Spearman rank correlation coefficients with complete linkage as distance measurement using Cluster v3.0 software [35,36]. Clusters were visualized in a heatmap using Java TreeView (http://jtreeview.sourceforge.net).

### Differential expression analyses

Transcript abundance values were compared among samples using the EdgeR package (bioconductor) to assess statistical significance [37]. The general linear models capability (glm EdgeR) was used to account for the multiple factors in the experiment (i.e. colony, developmental stage and irradiation treatment). The non-normalized expected counts for each gene, calculated by RSEM in the previous step, were used as input for EdgeR; contrasts were built between the three main overall effects and between the six pairwise comparisons of biological relevance for a total of nine comparisons. The data was normalized between each pair of groups or samples with the TMM method using the calcNormFactors function to account for the effect of RNA composition. Out of the 10,775 genes identified, a total of 9,920 genes were used for differential analyses after filtering out genes with count per million mapped reads less than 2 in any of the three replicate samples.

### Gene ontology term enrichment

The bioconductor package “topGO” was used to run a GO term enrichment analysis on the data [38]. Genes declared significantly differentially regulated on each of the treatment-wise comparisons and mapped with GO terms were used as predefined lists of genes of interest. Terms with less than 5 annotated genes were cut-out from the GO hierarchy during the enrichment using the nodSize option, and only the “biological processes” ontology was used. GO terms were then subjected to the classic Fisher’s exact test included in the topGO package.

### Availability of supporting data

All raw reads were submitted to the NCBI Sequence Read Archive under accession numbers SAMN02208095 – SAMN02208112 associated with BioProject PRJNA208956.

## Competing interests

The authors declare that they have no competing interests.

## Author’s contributions

Conceived and designed the experiments: SMG. Performed the experiments: SMG. Analyzed the data: BC, BH and SMG. Evaluated conclusions: BC, BH, SH and SMG. Contributed reagents/materials/analysis tools: BC, BH, SH, and SMG. Wrote the paper: BC, BH, and SMG. All authors read and approved the final manuscript.

## Supplementary Material

Additional file 1: Table S1 Abundance of TMM normalized reads on each protein family. Click here for file

Additional file 2: Figure S1 Sub-cluster of identified Pfams derived from Figure 2. Differences between adults and pupae libraries across both types of flies. Subcluster shows Pfams with higuer abundance in the adult stage. Click here for file

Additional file 3: Figure S2 Sub-cluster of identified Pfams derived from Figure 2. Differences between adults and pupae libraries across both types of flies. Subcluster shows Pfams with higher abundance in pupae stage. Click here for file

Additional file 4: Table S2 Top 30 enriched GO terms in adult vs. pupae. Click here for file

Additional file 5: Table S3 Top 30 enriched GO terms in Irradiated vs. non-irradiated. Click here for file

Additional file 6: Table S4 Top enriched GO terms in irradiated vs. non-irradiated Vienna7 pupae. Click here for file

Additional file 7: Table S5 Top enriched GO terms in irradiated vs. non-irradiated Vienna7 adults. Click here for file
