# Supplementary figures and images for: A genomic perspective to assessing quality of mass-reared SIT flies used in Mediterranean fruit fly (Ceratitis capitata) eradication in California
Source: BMC Genomics. 2014 Feb 5;15:98. doi: 10.1186/1471-2164-15-98 (PMC3923235; doi:10.1186/1471-2164-15-98)

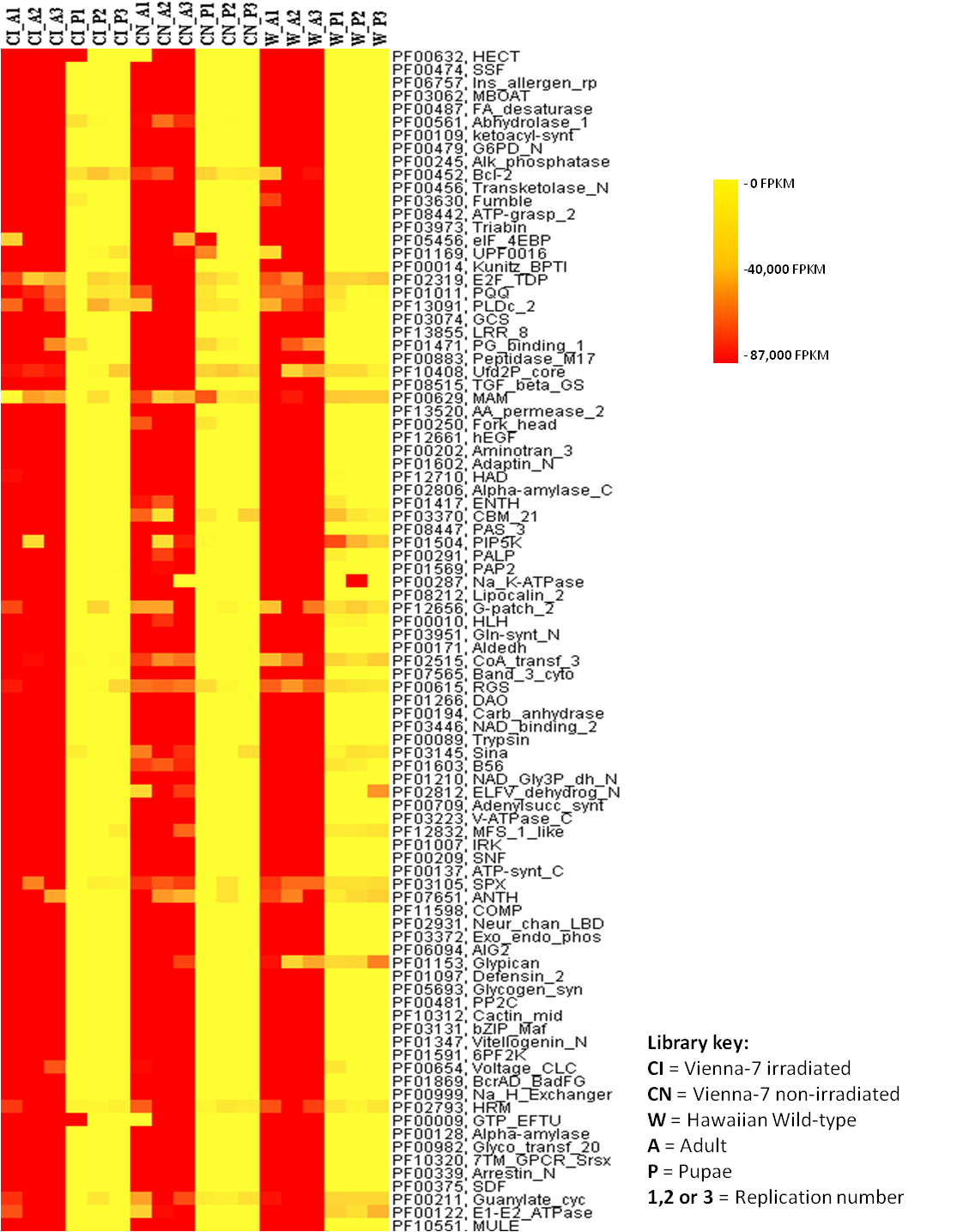

Supplement: Additional file 2: Figure S1 — Sub-cluster of identified Pfams derived from Figure 2. Differences between adults and pupae libraries across both types of flies. Subcluster shows Pfams with higuer abundance in the adult stage. [file 1471-2164-15-98-S2.docx]

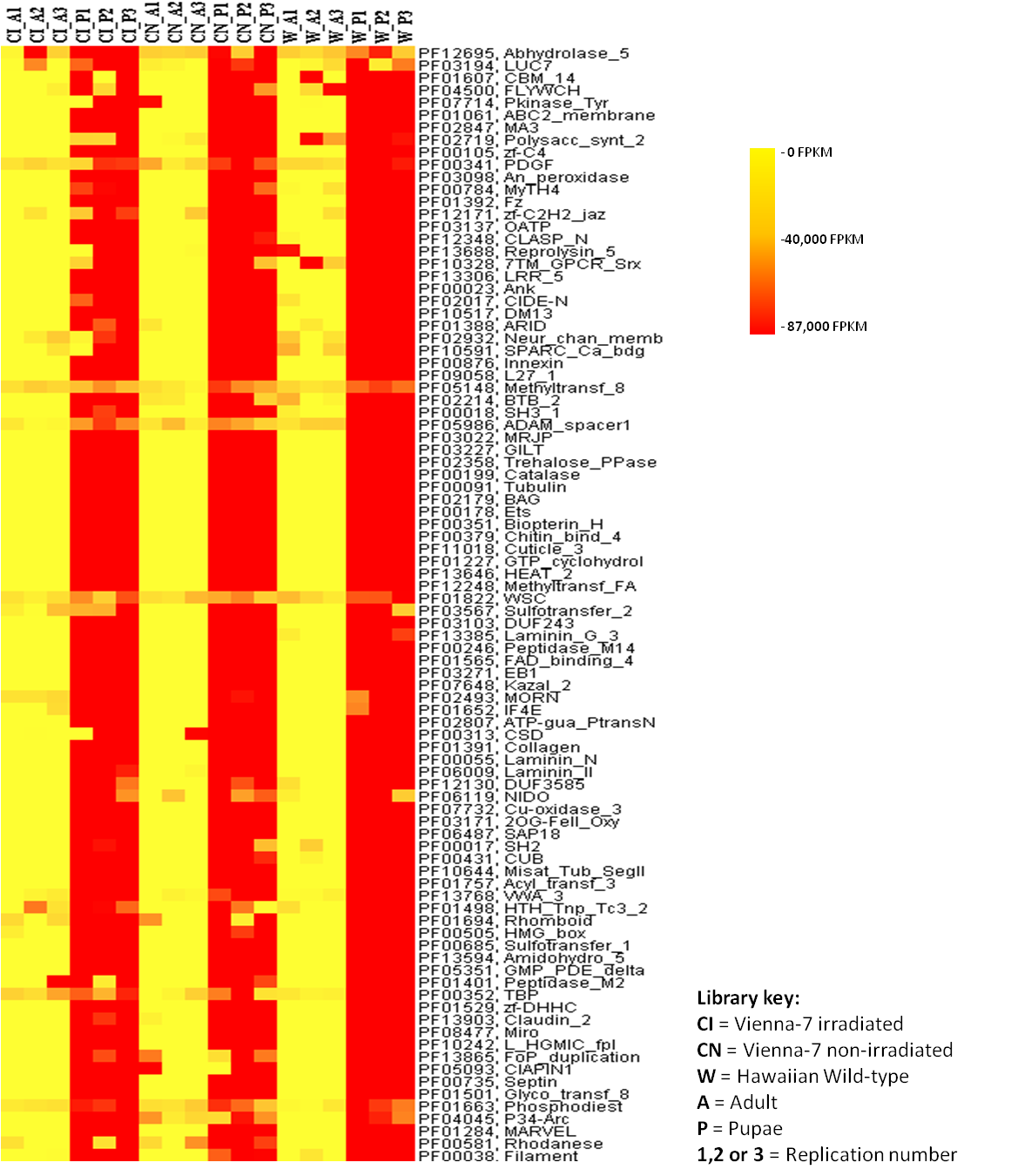

Supplement: Additional file 3: Figure S2 — Sub-cluster of identified Pfams derived from Figure 2. Differences between adults and pupae libraries across both types of flies. Subcluster shows Pfams with higher abundance in pupae stage. [file 1471-2164-15-98-S3.docx]
