# Supplementary material for: A genomic perspective to assessing quality of mass-reared SIT flies used in Mediterranean fruit fly (Ceratitis capitata) eradication in California
Source: BMC Genomics. 2014 Feb 5;15:98. doi: 10.1186/1471-2164-15-98 (PMC3923235; doi:10.1186/1471-2164-15-98)
Supplement: Additional file 4: Table S2 — Top 30 enriched GO terms in adult vs. pupae. [file 1471-2164-15-98-S4.docx]

**Additional file 4: Table S2.** Top 30 enriched GO terms in adult vs. pupae.

| Term | Annotated | Significant | Expected | Fisher exact test | p-value |
| --- | --- | --- | --- | --- | --- |
| *Adult enriched* | |  |  |  |  |
| GO:0044710 | single-organism metabolic process | 947 | 452 | 334.33 | 2.40E-19 |
| GO:0044281 | small molecule metabolic process | 699 | 349 | 246.77 | 2.40E-18 |
| GO:0006082 | organic acid metabolic process | 323 | 183 | 114.03 | 3.00E-16 |
| GO:0019752 | carboxylic acid metabolic process | 299 | 171 | 105.56 | 9.60E-16 |
| GO:0043436 | oxoacid metabolic process | 318 | 179 | 112.27 | 1.60E-15 |
| GO:1901564 | organonitrogen compound metabolic processes | 506 | 251 | 178.64 | 1.40E-12 |
| GO:0006520 | cellular amino acid metabolic process | 166 | 98 | 58.6 | 1.80E-10 |
| GO:0044711 | single-organism biosynthetic process | 178 | 103 | 62.84 | 3.00E-10 |
| GO:1901605 | alpha-amino acid metabolic process | 89 | 59 | 31.42 | 1.80E-09 |
| GO:0006629 | lipid metabolic process | 332 | 168 | 117.21 | 1.90E-09 |
| GO:0044283 | small molecule biosynthetic process | 165 | 94 | 58.25 | 5.80E-09 |
| GO:0005975 | carbohydrate metabolic process | 285 | 146 | 100.62 | 8.60E-09 |
| GO:0006811 | ion transport | 211 | 114 | 74.49 | 9.10E-09 |
| GO:0016053 | organic acid biosynthetic process | 116 | 70 | 40.95 | 2.20E-08 |
| GO:0046394 | carboxylic acid biosynthetic process | 116 | 70 | 40.95 | 2.20E-08 |
| GO:0044723 | single-organism carbohydrate metabolic processes | 184 | 101 | 64.96 | 2.30E-08 |
| GO:0032787 | monocarboxylic acid metabolic process | 121 | 71 | 42.72 | 9.00E-08 |
| GO:0005996 | monosaccharide metabolic process | 88 | 55 | 31.07 | 1.40E-07 |
| GO:0044282 | small molecule catabolic process | 97 | 59 | 34.24 | 2.00E-07 |
| GO:0044712 | single-organism catabolic process | 97 | 59 | 34.24 | 2.00E-07 |
| GO:0044255 | cellular lipid metabolic process | 242 | 123 | 85.44 | 2.50E-07 |
| GO:1901566 | organonitrogen compound biosynthetic processes | 185 | 98 | 65.31 | 3.80E-07 |
| GO:0044765 | single-organism transport | 659 | 289 | 232.65 | 4.90E-07 |
| GO:0006810 | transport | 852 | 362 | 300.79 | 7.10E-07 |
| GO:0007586 | digestion | 45 | 32 | 15.89 | 9.50E-07 |
| GO:0019318 | hexose metabolic process | 77 | 48 | 27.18 | 1.00E-06 |
| GO:0044724 | single-organism carbohydrate catabolic processes | 47 | 33 | 16.59 | 1.00E-06 |
| GO:0009308 | amine metabolic process | 48 | 33 | 16.95 | 2.20E-06 |
| GO:0051234 | establishment of localization | 868 | 365 | 306.44 | 2.30E-06 |
| GO:0008652 | cellular amino acid biosynthetic process | 52 | 35 | 18.36 | 2.30E-06 |

| ***Pupae enriched*** | |  |  |  |  |
| --- | --- | --- | --- | --- | --- |
| GO:0007155 | cell adhesion | 226 | 112 | 65.89 | 2.60E-11 |
| GO:0022610 | biological adhesion | 226 | 112 | 65.89 | 2.60E-11 |
| GO:0016337 | cell-cell adhesion | 85 | 51 | 24.78 | 2.30E-09 |
| GO:0007156 | homophilic cell adhesion | 34 | 25 | 9.91 | 1.00E-07 |
| GO:0048856 | anatomical structure development | 1301 | 451 | 379.33 | 1.20E-07 |
| GO:0007424 | open tracheal system development | 95 | 52 | 27.7 | 1.20E-07 |
| GO:0044767 | single-organism developmental process | 1185 | 415 | 345.51 | 1.40E-07 |
| GO:0009653 | anatomical structure morphogenesis | 761 | 279 | 221.88 | 4.70E-07 |
| GO:0016339 | calcium-dependent cell-cell adhesion | 19 | 16 | 5.54 | 9.50E-07 |
| GO:0060541 | respiratory system development | 116 | 58 | 33.82 | 1.40E-06 |
| GO:0048731 | system development | 1025 | 358 | 298.86 | 2.40E-06 |
| GO:0030036 | actin cytoskeleton organization | 151 | 70 | 44.03 | 3.90E-06 |
| GO:0030029 | actin filament-based process | 161 | 73 | 46.94 | 6.60E-06 |
| GO:0032502 | developmental process | 1511 | 502 | 440.56 | 9.50E-06 |
| GO:0051017 | actin filament bundle assembly | 27 | 19 | 7.87 | 1.10E-05 |
| GO:0060429 | epithelium development | 180 | 79 | 52.48 | 1.20E-05 |
| GO:0071822 | protein complex subunit organization | 243 | 101 | 70.85 | 1.40E-05 |
| GO:0035154 | terminal cell fate specification | 9 | 9 | 2.62 | 1.50E-05 |
| GO:0035152 | regulation of tube architecture | 34 | 22 | 9.91 | 1.70E-05 |
| GO:0044707 | single-multicellular organism process | 1699 | 556 | 495.37 | 1.80E-05 |
| GO:0048569 | post-embryonic organ development | 165 | 73 | 48.11 | 1.90E-05 |
| GO:0048563 | post-embryonic organ morphogenesis | 157 | 70 | 45.78 | 2.10E-05 |
| GO:0007476 | imaginal disc-derived wing morphogenesis | 119 | 56 | 34.7 | 2.10E-05 |
| GO:0007472 | wing disc morphogenesis | 122 | 57 | 35.57 | 2.40E-05 |
| GO:0007010 | cytoskeleton organization | 302 | 120 | 88.05 | 2.80E-05 |
| GO:0032989 | cellular component morphogenesis | 362 | 140 | 105.55 | 3.00E-05 |
| GO:0035220 | wing disc development | 142 | 64 | 41.4 | 3.10E-05 |
| GO:0007560 | imaginal disc morphogenesis | 156 | 69 | 45.48 | 3.30E-05 |
| GO:0007591 | molting cycle (chitin based cuticle) | 35 | 22 | 10.2 | 3.30E-05 |
| GO:0000902 | cell morphogenesis | 315 | 124 | 91.84 | 3.40E-05 |
