# Supplementary material for: A genomic perspective to assessing quality of mass-reared SIT flies used in Mediterranean fruit fly (Ceratitis capitata) eradication in California
Source: BMC Genomics. 2014 Feb 5;15:98. doi: 10.1186/1471-2164-15-98 (PMC3923235; doi:10.1186/1471-2164-15-98)
Supplement: Additional file 5: Table S3 — Top 30 enriched GO terms in Irradiated vs. non-irradiated. [file 1471-2164-15-98-S5.docx]

**Additional file 5: Table S3.** Top 30 enriched GO terms in Irradiated vs. non-irradiated

***Irradiated enriched***

| Term | Annotated | Significant | Expected | Fisher exact test | p-value |
| --- | --- | --- | --- | --- | --- |
| GO:0016126 | sterol biosynthetic process | 15 | 2 | 0.01 | 7.00E-05 |
| GO:0008299 | isoprenoid biosynthetic process | 16 | 2 | 0.02 | 8.00E-05 |
| GO:0006720 | isoprenoid metabolic process | 24 | 2 | 0.02 | 0.00018 |
| GO:0006694 | steroid biosynthetic process | 26 | 2 | 0.02 | 0.00021 |
| GO:0016125 | sterol metabolic process | 36 | 2 | 0.03 | 0.00041 |
| GO:0046165 | alcohol biosynthetic process | 36 | 2 | 0.03 | 0.00041 |
| GO:1901617 | organic hydroxy compound biosynthetic processes | 48 | 2 | 0.05 | 0.00074 |
| GO:0008202 | steroid metabolic process | 54 | 2 | 0.05 | 0.00094 |
| GO:0006066 | alcohol metabolic process | 111 | 2 | 0.1 | 0.00393 |
| GO:0006084 | acetyl-CoA metabolic process | 5 | 1 | 0 | 0.0047 |
| GO:1901615 | organic hydroxy compound metabolic processes | 151 | 2 | 0.14 | 0.00719 |
| GO:0008610 | lipid biosynthetic process | 160 | 2 | 0.15 | 0.00805 |
| GO:0044283 | small molecule biosynthetic process | 165 | 2 | 0.16 | 0.00855 |
| GO:0006695 | cholesterol biosynthetic process | 10 | 1 | 0.01 | 0.00939 |
| GO:0044711 | single-organism biosynthetic process | 178 | 2 | 0.17 | 0.00992 |
| GO:0006637 | acyl-CoA metabolic process | 13 | 1 | 0.01 | 0.01219 |
| GO:0035383 | thioester metabolic process | 13 | 1 | 0.01 | 0.01219 |
| GO:0044255 | cellular lipid metabolic process | 242 | 2 | 0.23 | 0.01798 |
| GO:0045132 | meiotic chromosome segregation | 24 | 1 | 0.02 | 0.02243 |
| GO:0006278 | RNA-dependent DNA replication | 27 | 1 | 0.03 | 0.0252 |
| GO:0008203 | cholesterol metabolic process | 30 | 1 | 0.03 | 0.02797 |
| GO:0006629 | lipid metabolic process | 332 | 2 | 0.31 | 0.0329 |
| GO:0015074 | DNA integration | 40 | 1 | 0.04 | 0.03717 |
| GO:0042493 | response to drug | 49 | 1 | 0.05 | 0.04538 |

***Non-irradiated enriched***

| GO:0019058 | viral infectious cycle | 18 | 2 | 0.01 | 5.10E-05 |
| --- | --- | --- | --- | --- | --- |
| GO:0022415 | viral reproductive process | 32 | 2 | 0.02 | 0.00016 |
| GO:0016032 | viral reproduction | 39 | 2 | 0.03 | 0.00025 |
| GO:0044764 | multi-organism cellular process | 39 | 2 | 0.03 | 0.00025 |
| GO:0044703 | multi-organism reproductive process | 113 | 2 | 0.08 | 0.00207 |
| GO:0034645 | cellular macromolecule biosynthetic proc... | 850 | 3 | 0.6 | 0.008 |
| GO:0009059 | macromolecule biosynthetic process | 873 | 3 | 0.62 | 0.00867 |
| GO:0019079 | viral genome replication | 13 | 1 | 0.01 | 0.00916 |
| GO:0090304 | nucleic acid metabolic process | 948 | 3 | 0.67 | 0.0111 |
| GO:0051704 | multi-organism process | 270 | 2 | 0.19 | 0.01158 |
| GO:0032196 | transposition | 17 | 1 | 0.01 | 0.01197 |
| GO:0006278 | RNA-dependent DNA replication | 27 | 1 | 0.02 | 0.01896 |
| GO:0006139 | nucleobase-containing compound metabolic... | 1166 | 3 | 0.82 | 0.02067 |
| GO:0044249 | cellular biosynthetic process | 1185 | 3 | 0.84 | 0.0217 |
| GO:1901576 | organic substance biosynthetic process | 1197 | 3 | 0.85 | 0.02236 |
| GO:0046483 | heterocycle metabolic process | 1212 | 3 | 0.86 | 0.02322 |
| GO:0009058 | biosynthetic process | 1219 | 3 | 0.86 | 0.02362 |
| GO:0006725 | cellular aromatic compound metabolic pro... | 1231 | 3 | 0.87 | 0.02433 |
| GO:1901360 | organic cyclic compound metabolic proces... | 1289 | 3 | 0.91 | 0.02793 |
| GO:0015074 | DNA integration | 40 | 1 | 0.03 | 0.028 |
| GO:0034641 | cellular nitrogen compound metabolic pro... | 1291 | 3 | 0.91 | 0.02806 |
