# Supplementary material for: A genomic perspective to assessing quality of mass-reared SIT flies used in Mediterranean fruit fly (Ceratitis capitata) eradication in California
Source: BMC Genomics. 2014 Feb 5;15:98. doi: 10.1186/1471-2164-15-98 (PMC3923235; doi:10.1186/1471-2164-15-98)
Supplement: Additional file 6: Table S4 — Top enriched GO terms in irradiated vs. non-irradiated Vienna7 pupae. [file 1471-2164-15-98-S6.docx]

**Additional file 6: Table S4.** Top enriched GO terms in irradiated vs. non-irradiated Vienna7 pupae.

***Irradiated enriched***

| Term | Annotated | Significant | Expected | Fisher exact test | p-value |
| --- | --- | --- | --- | --- | --- |
| GO:0006974 | response to DNA damage stimulus | 136 | 18 | 2.66 | 5.20E-11 |
| GO:0006281 | DNA repair | 82 | 13 | 1.6 | 3.50E-09 |
| GO:0006259 | DNA metabolic process | 239 | 19 | 4.67 | 8.90E-08 |
| GO:0006260 | DNA replication | 93 | 12 | 1.82 | 1.60E-07 |
| GO:0033554 | cellular response to stress | 276 | 19 | 5.4 | 8.70E-07 |
| GO:0006950 | response to stress | 556 | 27 | 10.87 | 3.10E-06 |
| GO:0042255 | ribosome assembly | 7 | 4 | 0.14 | 4.50E-06 |
| GO:0006302 | double-strand break repair | 25 | 5 | 0.49 | 9.90E-05 |
| GO:0090304 | nucleic acid metabolic process | 948 | 33 | 18.53 | 0.00023 |
| GO:0006919 | activation of cysteine-type endopeptidases | 7 | 3 | 0.14 | 0.00024 |
| GO:0097202 | activation of cysteine-type endopeptidases | 7 | 3 | 0.14 | 0.00024 |
| GO:0022618 | ribonucleoprotein complex assembly | 20 | 4 | 0.39 | 0.00052 |
| GO:0034641 | cellular nitrogen compound metabolism | 1291 | 39 | 25.24 | 0.00098 |
| GO:0042254 | ribosome biogenesis | 60 | 6 | 1.17 | 0.001 |
| GO:0042981 | regulation of apoptotic process | 133 | 9 | 2.6 | 0.00102 |
| GO:0010950 | positive regulation of endopeptidase activity | 11 | 3 | 0.22 | 0.00106 |
| GO:0010952 | positive regulation of peptidase activity | 11 | 3 | 0.22 | 0.00106 |
| GO:0043280 | positive regulation of cysteine-type endopeptidase | 11 | 3 | 0.22 | 0.00106 |
| GO:2001056 | positive regulation of cysteine-type endopeptidase | 11 | 3 | 0.22 | 0.00106 |
| GO:0070925 | organelle assembly | 41 | 5 | 0.8 | 0.00109 |

***Non-irradiated enriched***

| GO:0019079 | viral genome replication | 13 | 3 | 0.02 | 7.80E-07 |
| --- | --- | --- | --- | --- | --- |
| GO:0019058 | viral infectious cycle | 18 | 3 | 0.03 | 2.20E-06 |
| GO:0022415 | viral reproductive process | 32 | 3 | 0.05 | 1.30E-05 |
| GO:0016032 | viral reproduction | 39 | 3 | 0.06 | 2.40E-05 |
| GO:0044764 | multi-organism cellular process | 39 | 3 | 0.06 | 2.40E-05 |
| GO:0044703 | multi-organism reproductive process | 113 | 3 | 0.19 | 0.00059 |
| GO:0006508 | proteolysis | 423 | 4 | 0.7 | 0.00266 |
| GO:0051704 | multi-organism process | 270 | 3 | 0.45 | 0.00735 |
| GO:0030282 | bone mineralization | 9 | 1 | 0.01 | 0.01475 |
| GO:0031214 | biomineral tissue development | 10 | 1 | 0.02 | 0.01638 |
| GO:0006817 | phosphate ion transport | 17 | 1 | 0.03 | 0.02771 |
| GO:0019048 | virus-host interaction | 18 | 1 | 0.03 | 0.02932 |
| GO:0051701 | interaction with host | 20 | 1 | 0.03 | 0.03253 |
| GO:0044403 | symbiosis, mutualism | 25 | 1 | 0.04 | 0.04052 |
| GO:0044419 | interspecies interaction between organism | 25 | 1 | 0.04 | 0.04052 |
| GO:0001503 | ossification | 26 | 1 | 0.04 | 0.04211 |
| GO:0022414 | reproductive process | 526 | 3 | 0.87 | 0.0451 |
| GO:0015698 | inorganic anion transport | 32 | 1 | 0.05 | 0.05161 |
| GO:0000003 | reproduction | 564 | 3 | 0.93 | 0.05403 |
| GO:0019538 | protein metabolic process | 982 | 4 | 1.62 | 0.05441 |
