# Supplementary material for: A genomic perspective to assessing quality of mass-reared SIT flies used in Mediterranean fruit fly (Ceratitis capitata) eradication in California
Source: BMC Genomics. 2014 Feb 5;15:98. doi: 10.1186/1471-2164-15-98 (PMC3923235; doi:10.1186/1471-2164-15-98)
Supplement: Additional file 7: Table S5 — Top enriched GO terms in irradiated vs. non-irradiated Vienna7 adults. [file 1471-2164-15-98-S7.docx]

**Additional file 7: Table S5.** Top enriched GO terms in irradiated vs. non-irradiated Vienna7 adults.

***Irradiated enriched***

| Term | Annotated | Significant | Expected | Fisher exact test | p-value |
| --- | --- | --- | --- | --- | --- |
| GO:0001539 | ciliary or flagellar motility | 11 | 6 | 0.32 | 2.10E-07 |
| GO:0006595 | polyamine metabolic process | 8 | 5 | 0.23 | 9.80E-07 |
| GO:0009309 | amine biosynthetic process | 14 | 5 | 0.41 | 3.10E-05 |
| GO:0019752 | carboxylic acid metabolic process | 299 | 22 | 8.66 | 3.40E-05 |
| GO:0015672 | monovalent inorganic cation transport | 90 | 11 | 2.61 | 4.60E-05 |
| GO:0009308 | amine metabolic process | 48 | 8 | 1.39 | 5.60E-05 |
| GO:0006520 | cellular amino acid metabolic process | 166 | 15 | 4.81 | 7.10E-05 |
| GO:1901564 | organonitrogen compound metabolism | 506 | 30 | 14.66 | 7.20E-05 |
| GO:0043436 | oxoacid metabolic process | 318 | 22 | 9.21 | 8.80E-05 |
| GO:0006811 | ion transport | 211 | 17 | 6.11 | 9.80E-05 |
| GO:0044281 | small molecule metabolic process | 699 | 37 | 20.25 | 9.90E-05 |
| GO:0006814 | sodium ion transport | 52 | 8 | 1.51 | 0.0001 |
| GO:0006082 | organic acid metabolic process | 323 | 22 | 9.36 | 0.00011 |
| GO:0007018 | microtubule-based movement | 53 | 8 | 1.54 | 0.00012 |
| GO:0006596 | polyamine biosynthetic process | 5 | 3 | 0.14 | 0.00023 |
| GO:0006812 | cation transport | 146 | 13 | 4.23 | 0.00026 |
| GO:0046034 | ATP metabolic process | 47 | 7 | 1.36 | 0.00035 |
| GO:0006200 | ATP catabolic process | 34 | 6 | 0.98 | 0.00036 |
| GO:0008215 | spermine metabolic process | 6 | 3 | 0.17 | 0.00045 |
| GO:0042401 | cellular biogenic amine biosynthetic processes | 6 | 3 | 0.17 | 0.00045 |

***Down-regulated genes***

| GO:0009112 | nucleobase metabolic process | 23 | 5 | 0.55 | 0.00017 |
| --- | --- | --- | --- | --- | --- |
| GO:0006546 | glycine catabolic process | 6 | 3 | 0.14 | 0.00025 |
| GO:0009071 | serine family amino acid catabolic proceses | 6 | 3 | 0.14 | 0.00025 |
| GO:0046112 | nucleobase biosynthetic process | 6 | 3 | 0.14 | 0.00025 |
| GO:0006144 | purine nucleobase metabolic process | 16 | 4 | 0.38 | 0.00044 |
| GO:0006544 | glycine metabolic process | 9 | 3 | 0.21 | 0.00099 |
| GO:0044710 | single-organism metabolic process | 947 | 36 | 22.53 | 0.00138 |
| GO:0006629 | lipid metabolic process | 332 | 17 | 7.9 | 0.00185 |
| GO:1901606 | alpha-amino acid catabolic process | 40 | 5 | 0.95 | 0.00234 |
| GO:0009127 | purine nucleoside monophosphate biosynthesis | 13 | 3 | 0.31 | 0.00314 |
| GO:0009168 | purine ribonucleoside monophosphate biosinthesis | 13 | 3 | 0.31 | 0.00314 |
| GO:0006030 | chitin metabolic process | 27 | 4 | 0.64 | 0.00348 |
| GO:0009063 | cellular amino acid catabolic process | 44 | 5 | 1.05 | 0.00359 |
| GO:0009126 | purine nucleoside monophosphate metabolism | 14 | 3 | 0.33 | 0.00393 |
| GO:0009167 | purine ribonucleoside monophosphate metabolism | 14 | 3 | 0.33 | 0.00393 |
| GO:0016054 | organic acid catabolic process | 67 | 6 | 1.59 | 0.00482 |
| GO:0046395 | carboxylic acid catabolic process | 67 | 6 | 1.59 | 0.00482 |
| GO:0016126 | sterol biosynthetic process | 15 | 3 | 0.36 | 0.00483 |
| GO:1901605 | alpha-amino acid metabolic process | 89 | 7 | 2.12 | 0.00488 |
